# Infection Without Borders

**DOI:** 10.1111/jpc.70349

**Published:** 2026-03-09

**Authors:** Jessie T. Lu, Karen Cheung, Deshan F. Sebaratnam

**Affiliations:** ^1^ School of Clinical Medicine University of New South Wales Medicine and Health Sydney New South Wales Australia; ^2^ Department of Dermatology Liverpool Hospital Liverpool New South Wales Australia; ^3^ The Skin Hospital Sydney New South Wales Australia; ^4^ Department of Anatomical Pathology Douglass Hanly Moir Pathology Sydney New South Wales Australia

## 1 Infection Without Borders

**FIGURE 1:**
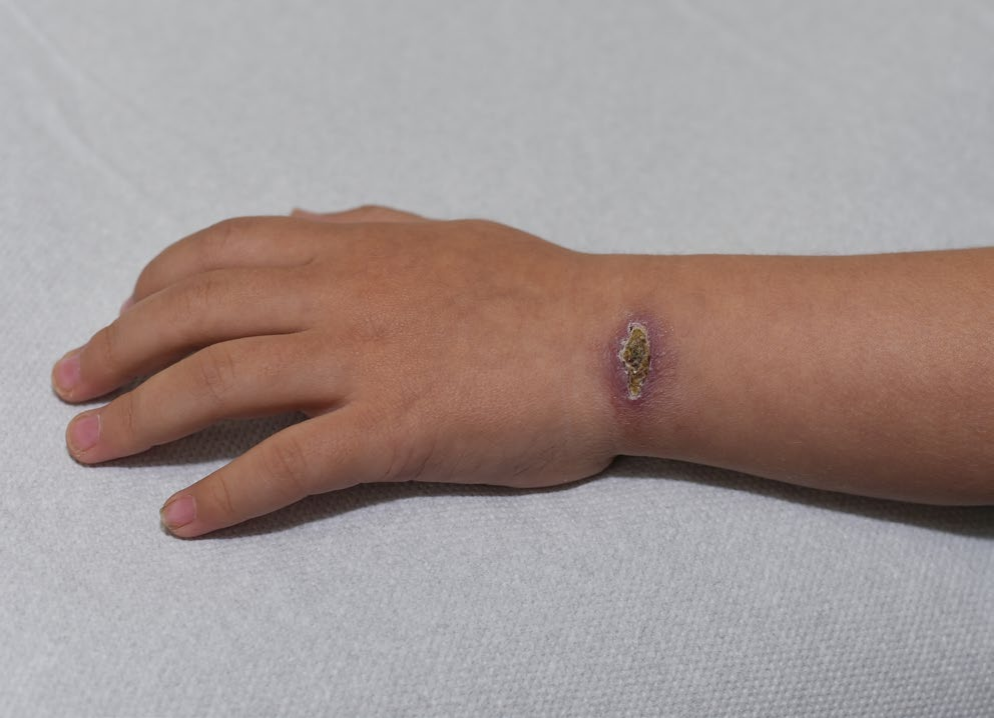
Non‐healing crusted violaceous plaque of the left wrist.

A 3‐year‐old female presented to Dermatology with her parents for review of a non‐healing crusted violaceous plaque on her wrist which had been present for 12 months (Figure 1). The onset of the lesion coincided with the family's immigration from Kabul, Afghanistan, where the patient had resided since birth. The plaque was tender, but the patient was otherwise well with no fever, fatigue, weight loss, lymphadenopathy, hepatosplenomegaly, mucosal ulceration or skin lesions elsewhere. The parents did not recall any exposure to animals, insect bites or trauma.

**FIGURE 2:**
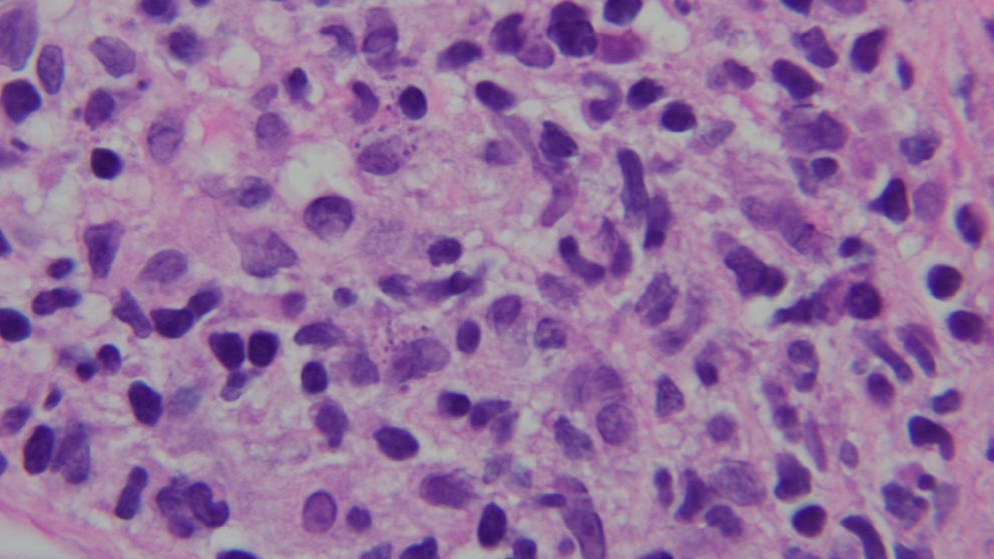
High‐power H&E stained section showing intracytoplasmic round bodies within histiocytes.

A punch biopsy was performed which demonstrated intracytoplasmic round bodies within multiple histiocytes on a background of a mixed inflammatory infiltrate composed of lymphocytes, plasma cells and histiocytes (Figure 2). CD1a staining was positive in these round bodies.

What is your diagnosis?

## 2 Discussion

The clinical and histopathological features in this case are in keeping with cutaneous leishmaniasis.

Leishmaniasis is an infection carried by protozoa of the *Leishmania* genus, of which there are over 20 species. These parasites are transmitted to humans by the bite of infected female sandflies of the *Phlebotomus* (old world) or *Lutzomyia* (new world) genera and cause three distinct forms of the disease: visceral leishmaniasis (kala‐azar), mucocutaneous leishmaniasis and the more common cutaneous leishmaniasis. The World Health Organisation recognises leishmaniasis as a neglected tropical disease which presently poses a major health problem in the Americas, East Africa, North Africa and West and South‐East Asia [1]. The paediatric population experiences a significant burden of the disease with the peak incidence in children aged 5–10 years [2]. In Australia, there have been no reported autochthonous cases of leishmaniasis to date although imported cases are not infrequent and have been rising along with global travel and migration [3].

Classically, cutaneous leishmaniasis presents as either a solitary or few erythematous papules at an exposed site, such as the face or limbs. These evolve into painless ulcers, which often heal without intervention, leaving a depressed scar [4]. Importantly, the incubation period of the infection can be anywhere from a week to over a year for other species. This underscores the importance of a thorough travel history and high clinical suspicion for leishmaniasis in returned travellers, immigrants and military personnel. Patients from endemic regions may already have some familiarity with the infection, and may even be the first to consider the diagnosis, such as in this case, where the patient's father demonstrated a scar from previous infection when prompted.

The clinical manifestations of cutaneous leishmaniasis are non‐specific and can mimic a variety of other infections, insect bites or tumours [5]. Dermoscopy may reveal features such as keratin plugging or parakeratotic hyperkeratosis [6]. A key histological feature is the visualisation of amastigotes, or Leishman–Donovan bodies [5]. These are small, round bodies, 2–4 μm in diameter, usually visible as blue dots within macrophages using a Giesma stain [4]. The diagnostic sensitivity of histopathology in Old World cutaneous leishmaniasis, however, is only about 60% and does not enable species identification [5]. A diagnosis could be made in this case based on histopathology, due to visualisation of amastigotes; however, these are only observed in an estimated 36% of cases in H&E staining [7], and CD1a positivity. The gold standard for diagnosis of cutaneous leishmaniasis is polymerase chain reaction on fresh or formalin‐fixed tissue [3], which is not only highly sensitive, but also enables accurate speciation; however, accessibility may be a barrier. PCR may be indicated in cases of diagnostic uncertainty or prior to active intervention as the therapeutic approach taken may be dictated by the species.

## 3 Learning Points

This case reminds clinicians in non‐endemic settings to remain vigilant for cases of leishmaniasis infection. While histopathology and molecular testing are important for diagnostic confirmation, early recognition of clinical presentation and a thorough travel history to characterise the geography of infection are crucial for timely diagnosis and management.

## Funding

The authors have nothing to report.

## Ethics Statement

The authors declare that the research presented in this manuscript adheres to the ethical principles outlined by the South Western Sydney Local Health District Research Ethics Committee. All procedures involving human participants were conducted in accordance with the ethical standards of South Western Sydney Local Health District and the Declaration of Helsinki (1964), as revised in 2013.

## Consent

The parents of the patient have provided written consent for the use of their child's de‐identified clinical images in this work.

## Conflicts of Interest

D.F.S. has received consulting or speaking fees from Novartis, AbbVie, Janssen, Pfizer, Galderma, Leo Pharmacy, Amgen, Bristol Myers Squibb, Viatrus, Mayne Pharmaceuticals, Johnson & Johnson and Neutrogena. J.T.L. and K.C. have no conflicts of interest to disclose.

## Data Availability

Data sharing not applicable to this article as no datasets were generated or analysed during the current study.
